# Towards targeting protein-protein interfaces with small molecules

**DOI:** 10.1186/1758-2946-3-S1-O21

**Published:** 2011-04-19

**Authors:** Holger Gohlke, Alexander Metz, Christopher Pfleger, Dennis M Krüger, Sina Kazemi

**Affiliations:** 1 Department of Mathematics and Natural Sciences, Heinrich-Heine-University, 40225 Düsseldorf, Germany

## 

A promising way to interfere with biological processes is through the control of protein-protein interactions by means of small molecules that modulate the formation of protein-protein complexes. Although the feasibility of this approach has been demonstrated in principle by recent results, many of the small-molecule modulators known to date have not been found by rational design approaches. In large part this is due to the challenges that one faces in dealing with protein binding epitopes compared to, e.g., enzyme binding pockets.

Recent advances in the understanding of the energetics and dynamics of protein binding interfaces[1] and methodological developments in the field of structure-based drug design methods may open up a way to apply rational design approaches also for finding protein-protein interaction modulators.2 Here, we first show in a retrospective analysis of the well-investigated interleukin-2 system how I) potential binding sites in an interface can be identified from an unbound protein structure, II) the interface can be dissected in terms of energetic contributions of single residues, and III) one can make use of this knowledge for guiding the development of small-molecule modulators. When applied to a leukaemia-associated fusion protein in a prospective manner, the predictive character of the methodology is demonstrated [2].

Another challenge arises from the fact that protein-protein interfaces are flexible. In the second part, we thus demonstrate a novel approach for including protein flexibility into protein-ligand docking[3]. This approach is based on elastic potential grids, which provide an accurate and efficient representation of intermolecular interactions in fully-flexible docking.
